# Atividades de Vida Diária, Atividade Física, Aptidão Física e Qualidade de Vida em Crianças com Cardiopatia Congênita: Um Estudo de Caso-Controle

**DOI:** 10.36660/abc.20230022

**Published:** 2023-09-27

**Authors:** Berfin Kişin, Sema Savci, Buse Ozcan Kahraman, Aylin Tanriverdi, Hazer Erçan Bozyer, Halise Zeynep Genç, Mustafa Kir

**Affiliations:** 1 Dokuz Eylül University Health Science Institute Izmir Turquia Dokuz Eylül University – Health Science Institute, Izmir – Turquia; 2 Acıbadem University Department of Physiotherapy and Rehabilitation Istanbul Turquia Acıbadem University - Department of Physiotherapy and Rehabilitation, Istanbul - Turquia; 3 Dokuz Eylül University School of Physical Therapy and Rehabilitation Izmir Turquia Dokuz Eylül University – School of Physical Therapy and Rehabilitation, Izmir – Turquia; 4 Çankırı Karatekin University Department of Physiotherapy and Rehabilitation Çankırı Turquia Çankırı Karatekin University - Department of Physiotherapy and Rehabilitation, Çankırı – Turquia; 5 Dokuz Eylül University Department of Pediatric Cardiology Izmir Turquia Dokuz Eylül University – Department of Pediatric Cardiology, Izmir – Turquia; 6 Başakşehir Çam and Sakura City Hospital Department of Pediatric Cardiology Istanbul Turquia Başakşehir Çam and Sakura City Hospital – Department of Pediatric Cardiology, Istanbul – Turquia

**Keywords:** Cardiopatias Congênitas, Aptidão Física, Atividade Motora, Atividades Cotidianas, Qualidade de Vida, Estudos de Casos e Controles

## Abstract

**Fundamento:**

Apesar dos relatos de redução da aptidão física em crianças com cardiopatia congênita (CC), não foram realizadas avaliações específicas de desempenho para atividades de vida diária.

**Objetivos:**

O objetivo foi comparar as atividades de vida diária, qualidade de vida, postura, aptidão física e níveis de atividade física entre crianças com CC e controles saudáveis (CS).

**Métodos:**

O estudo incluiu 30 crianças, de 6 a 14 anos, com diagnóstico de CC moderada ou grave e 30 consideradas CS pareadas por idade e sexo. Os dados sociodemográficos e clínicos dos participantes foram registrados. Todos os participantes realizaram diversos testes: teste de TGlittre-P para atividades de vida diária; teste de caminhada de 6 minutos (TC6M) para capacidade funcional; bateria de testes Fitnessgram para aptidão física; dinamômetro de mão para medir a força de preensão; pedômetro para medir a atividade física; além disso, a criança e os pais completaram o *Pediatric Quality of Life Inventory* (PedsQL) para avaliação da qualidade de vida, além de análises posturais. Valores de p < 0,05 foram considerados estatisticamente significativos.

**Resultados:**

Indivíduos com CC apresentaram um tempo de conclusão do teste TGlittre-P mais longo e uma distância de TC6M mais curta em comparação com o CS (TGlittre-P: CC 3,45 [3,24-4,02] min vs. CS 3,10 [2,57-3,23] min, TC6M: CC 514,00 [412,50-566,00] m vs. CS 591,50 [533,00-631,00] m). Para o grupo CC, os resultados dos testes de *sit-ups*, flexões, elevação do tronco e sentar e alcançar, dentro da bateria do Fitnessgram, além de força de preensão, postura e qualidade de vida foram menores do que os do grupo CS. Os níveis de atividade física foram semelhantes entre os grupos.

**Conclusões:**

O desempenho das atividades de vida diária, a capacidade funcional, a aptidão física, a postura e a qualidade de vida de crianças com CC moderada e grave foram afetados em comparação com seus pares saudáveis.

## Figura Central

**Figure:**
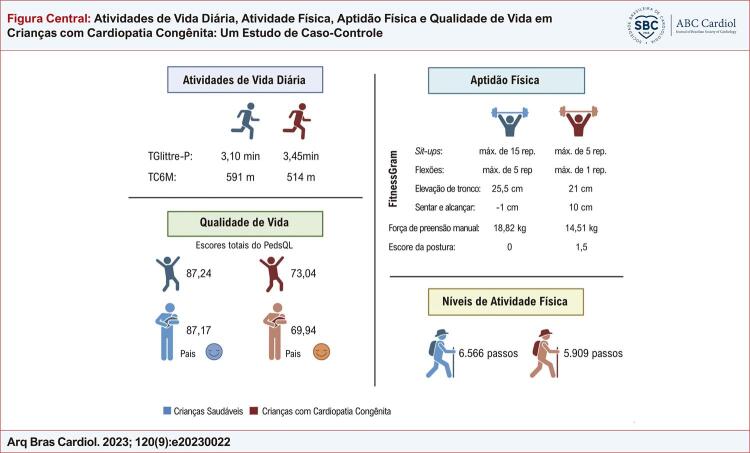
: Atividades de Vida Diária, Atividade Física, Aptidão Física e Qualidade de Vida em Crianças com Cardiopatia Congênita: Um Estudo de Caso-Controle

Atividades de vida diária, atividade física, aptidão física e qualidade de vida em crianças saudáveis e crianças com cardiopatia congênita.

## Introdução

As cardiopatias congênitas abrangem uma ampla variedade de defeitos cardíacos, de comprometimentos menores, não percebidos até a idade adulta ou detectados durante exercícios vigorosos, até malformações cardiovasculares graves que podem ser fatais. Estudos documentaram reduções na capacidade funcional, nas habilidades motoras e na força muscular periférica em crianças com CC complexa devido a causas multifatoriais, como cianose, aumento da circulação pulmonar, intervenções cardíacas e diagnósticos múltiplos.^1-3^

A aptidão física e os níveis de atividade física são importantes preditores da saúde cardiovascular. Uma aptidão física aprimorada nos estágios iniciais da vida está associada a um perfil cardiovascular mais saudável na idade adulta.^4^ Embora poucos estudos avaliem a aptidão física em crianças com CC,^3,5,6^ os níveis de atividade física diferem entre os estudos.^7-10^

Baixos níveis de atividade física e sedentarismo foram associados a defeitos posturais.^11^ Além disso, a resistência cardiovascular, a resistência muscular, a força muscular, a flexibilidade e a composição corporal, que são parâmetros de aptidão física, são componentes necessários para a obtenção da postura ideal. Estudos na literatura mostraram o desenvolvimento frequente de escoliose e deformidades cifóticas após a realização de esternotomias medianas em crianças com CC.^12,13^ No entanto, embora intervenções cirúrgicas sejam frequentemente realizadas em crianças com CC moderada e grave, os estudos que avaliam a postura são muito limitados.^14^

Os avanços no setor de saúde aumentaram a expectativa de vida dos indivíduos com CC e, portanto, a importância da qualidade de vida também aumentou. Estudos que avaliam a qualidade de vida relacionada à saúde em pacientes com CC enfatizam que a qualidade de vida relacionada à saúde física diminui devido à gravidade da doença e o número de intervenções cirúrgicas nesses indivíduos, em comparação com indivíduos saudáveis.^3,5,15,16^

A diminuição da capacidade funcional e da aptidão física pode afetar as atividades de vida diária. Um estudo avaliou as atividades de vida diária (AVD) de crianças com CC complexa.^17^ No entanto, o referido estudo utilizou apenas um questionário, em vez de um teste de desempenho específico, para a avaliação de AVD. Diante de todos esses fatores, nosso estudo objetivou comparar as atividades de vida diária, a qualidade de vida, a postura, a aptidão física e os níveis de atividade física entre crianças com CC e crianças saudáveis (CS).

## Métodos

Trata-se de um estudo de caso-controle em crianças com cardiopatia congênita (CC). O protocolo do estudo foi aceito pelo Comitê de Ética em Pesquisa Não Intervencionista da Universidade de Dokuz Eylül, com o número de aprovação 2020/29-58, em 12/07/2020. O estudo foi realizado entre novembro de 2020 e maio de 2022 no Hospital Universitário de Dokuz Eylül, Departamento de Pediatria, Divisão de Cardiologia Pediátrica. O consentimento informado por escrito foi obtido de todos os participantes e seus responsáveis.

O grupo CC incluiu 30 crianças com quadro clínico estável, com idades entre 6 e 14 anos,^18^ com diagnóstico de CC moderada ou grave (Defeito do Septo Atrial, Defeito do Septo Ventricular, Defeito do Septo Atrioventricular, Persistência do Canal Arterial, Coarctação da Aorta, Insuficiência Aórtica, Tetralogia de Fallot, Transposição das Grandes Artérias, Dupla Via de Saída do Ventrículo Direito, Retorno Venoso Pulmonar Anômalo Total, Ventrículo Único, Atresia Pulmonar, Estenose Pulmonar, Atresia Mitral, Dextrocardia). Utilizando o estudo de Warnes et al.,^19^ crianças com CC moderada a grave foram incluídas em nosso estudo e defeitos cardíacos menores isolados que não necessitaram de intervenção cirúrgica foram excluídos. Problemas ortopédicos ou neurológicos que afetassem os testes, distúrbios mentais ou psicológicos, infecção aguda ou fadiga geral, cirurgia cardíaca nos últimos seis meses e recusa em participar do estudo foram os critérios de exclusão utilizados. As crianças do grupo controle saudável foram selecionadas entre indivíduos saudáveis que se inscreveram no Departamento de Pediatria do Hospital Universitário de Dokuz Eylül e não haviam sido diagnosticados com qualquer doença. Trinta crianças saudáveis voluntárias de faixa etária e sexo semelhantes, não atletas, foram incluídas no estudo.

### Avaliação das Atividades de Vida Diária

O teste TGlittre-P é a versão pediátrica do teste original de Glittre-ADL desenvolvido para avaliar as atividades de vida diária dos indivíduos. O teste foi considerado válido e confiável para crianças saudáveis de 6 a 14 anos.^18^

O fisioterapeuta explicou o teste para cada criança, em seguida o mostrava e pedia para que experimentassem. Esse teste de desempenho exigia que a criança completasse cinco voltas no menor tempo carregando uma mochila,^18^ com peso entre 0,5 kg e 2,5 kg, determinado de acordo com idade e sexo do indivíduo. O teste iniciava-se quando a criança se levantava de uma cadeira com a sola dos pés tocando o solo. Em seguida, caminhavam por 5 metros, subiam e desciam dois lances de escada (17 cm de altura e 27 cm de largura) e caminhavam mais 5 metros. Três pinos de boliche coloridos, pesando 0,5 kg, foram retirados um a um da prateleira, ajustados na altura dos olhos pela criança, e recolocados na prateleira enquanto eram ajustados de acordo com o nível do umbigo, depois no chão, na prateleira no nível do umbigo e, finalmente, de volta à prateleira no nível dos olhos. Voltando do mesmo percurso e sentando na cadeira, a bateria de exercícios era concluída, e a próxima era iniciada imediatamente. Durante as cinco rodadas, as crianças moviam os pinos de boliche com uma das mãos à escolha delas enquanto um oxímetro de pulso era acoplado ao dedo indicador da outra mão. Durante o teste, as crianças receberam comandos verbais padrão, como “sente-se”, “levante-se” e “continue”. O tempo esperado do teste desenvolvido por Martins et al.,^20^ foi calculado e comparado com os dados de desempenho do teste das crianças.

### Avaliação da Capacidade Funcional

O teste de caminhada de 6 minutos (TC6M) foi utilizado para avaliar a capacidade de exercício funcional. Solicitou-se às crianças que caminhassem o mais rapidamente possível por um corredor de 30 m de comprimento, usando comandos verbais padrão como “você está indo muito bem” e “continue assim” ao final de cada minuto. Antes e após o teste, a frequência cardíaca, a saturação periférica de oxigênio e a distância percorrida foram registradas. Além disso, calculou-se a distância esperada de caminhada, conforme relatada por Geiger et al.,^21^ comparando-a com os dados de desempenho no teste das crianças.

### Avaliação da Aptidão Física

Os testes de *sit-ups*, flexões, elevação do tronco e sentar e alcançar, da bateria de testes Fitnessgram,^22,23^ foram utilizados para avaliar a aptidão física das crianças. O número máximo de repetições concluídas corretamente foi medido para os testes de flexões e *sit-ups*. A distância na posição de teste foi medida e registrada em centímetros para os testes de elevação de tronco e de sentar e alcançar.

### Avaliação da Força de Preensão Manual

A força de preensão manual foi avaliada por meio do dinamômetro de mão Jamar (Modelo 5030J1, Sammons Preston Rolyan, Bolingbrook, IL, EUA).^24^ As crianças estavam sentadas com as costas eretas. Pediu-se que elas apertassem o dinamômetro de mão com o máximo de força possível, com o cotovelo em 90° de flexão, o braço próximo ao corpo e o punho em posição neutra. As medições foram repetidas três vezes para as mãos direita e esquerda, com intervalo de 15 segundos, sendo os maiores valores registrados em quilogramas.

### Avaliação Postural

A ficha de análise postural de Corbin et al.,^25^ foi utilizada para determinar distúrbios posturais. O formulário baseia-se na identificação de distúrbios posturais com observação posterior e lateral, além de classificação de acordo com a gravidade (0, nenhum; 1, leve; 2, moderado; 3, grave). Ele também permite classificar o estado postural de acordo com o escore total. As avaliações foram realizadas na posição ortostática, sem sapatos e com roupas leves e confortáveis, adequadas para a avaliação da postura, e os achados apurados foram registrados.

### Avaliação da Atividade Física

Os níveis de atividade física das crianças foram avaliados por meio de um pedômetro (Yamax CW700 Digi-walker Pedometer, Yamax Corp, Tóquio, Japão).^26^ Os dispositivos foram usados para registrar o comprimento normal da passada e o peso corporal das crianças. Pedômetros foram fixados em suas roupas, determinando-se a projeção do ponto médio da patela até a superfície anterior da pelve. As crianças foram solicitadas a usar o pedômetro continuamente por sete dias, exceto ao tomar banho e dormir. Após sete dias de uso, uma contagem média de passos em um dia foi calculada.

### Avaliação da Qualidade de Vida

O PedsQL é uma escala de 23 itens que inclui função física, emocional, social e escolar.^27^ Escores mais altos indicam melhor qualidade de vida relacionada à saúde da criança. O formulário PedsQL 4.0 Generic Core Scales,^27^ para crianças de 5-7, 8-12 e 13-18 anos de idade e seus pais, foram usados em nosso estudo. O escore total da escala, o escore de função física e o escore de saúde psicossocial foram calculados, consistindo em escores de funcionalidade escolar, social e emocional.

Foi explicado às crianças que deveriam dizer se sentiam tontura, palpitações, dor no peito, dificuldade para respirar ou cansaço excessivo durante todos os testes. Elas poderiam descansar e continuar ou encerrar o teste, conforme necessário.

## Métodos Estatísticos

Com base no estudo que compara o teste TGlittre-ADL em pacientes com doença pulmonar obstrutiva crônica e um grupo controle de indivíduos saudáveis, o menor tamanho de amostra foi calculado usando o programa G*Power 3.1.^28^ Calculou-se que 36 participantes, incluindo pelo menos 18 crianças em cada grupo, devem ser incluídos no estudo, com um tamanho de efeito calculado de 0,97, probabilidade de erro alfa de 0,05 e um poder de 80%.

O programa IBM SPSS Statistics (Versão 26.0) foi utilizado para analisar os dados obtidos dos participantes. Foram usados valores de curtose/assimetria, testes de Shapiro-Wilk, gráfico Q-Q normal sem tendência e gráficos de histograma para determinar a conformidade das variáveis com a distribuição normal. A diferença entre as variáveis categóricas foi analisada pelo teste qui-quadrado. O teste de Mann-Whitney U foi usado para comparar as diferenças entre os grupos para condições que não se conformavam com a distribuição normal. Um teste t para grupos independentes foi usado para comparar as diferenças entre os grupos para condições que se conformavam à distribuição normal. As variáveis categóricas foram expressas em valores absolutos e percentuais. Variáveis contínuas com distribuição normal foram expressas como média e desvio padrão, e variáveis contínuas com distribuição anormal foram expressas como mediana e intervalo interquartílico. Valores de p < 0,05 foram considerados estatisticamente significativos.

## Resultados

As características demográficas dos participantes são apresentadas na Tabela 1. Não houve diferença estatisticamente significativa entre o grupo CC e o grupo controle em termos de idade, sexo e altura. O peso corporal e o índice de massa corporal do grupo CC foram menores do que o grupo controle. Todas as crianças foram obrigadas a frequentar a escola remotamente devido às restrições impostas pela pandemia da COVID-19 durante o período em que foram incluídas no estudo. Consequentemente, não há diferença entre o índice de frequência escolar das crianças.

**Tabela 1:** – Características demográficas dos participantes

Parâmetro	Grupo CC (n = 30)	Grupo controle (n = 30)	Valor de x^2^ /z	Valor de p
Sexo Menina/Menino (%)	11/19 (36,7/63,3)	11/19 (36,7/63,3)	0,000 ^a^	1,000
Idade (anos)	9,50 (8,00-12,25)	9,50 (8,00-13,00)	-0,037 ^b^	0,970
Altura (cm)	134,50 (128,50-145,00)	137,00 (130,00-149,25)	-0,710 ^b^	0,477
Peso (kg)	27,50 (25,00-45,00)	36,50 (27,75-52,25)	-2,058 ^b^	0,040*
IMC (kg/m^2^)	16,18 (14,11-20,26)	19,87 (16,71-22,06)	-2,728 ^b^	0,006**

a: Teste qui-quadrado de Yates; b: Teste Mann Whitney U; IMC: índice de massa corporal; CC: cardiopatia congênita, p < 0,05*, p < 0,01**

As características clínicas do grupo CC são apresentadas na Tabela 2.

**Tabela 2:** – Características clínicas do grupo CC

Tipo de CC	
Acianótica	20 (66,6%)
Cianótica	10 (33,3%)
Gravidade da CC*	
Moderada	16 (53,3%)
Grave	14 (46,6%)
**Tratamento cirúrgico**	
Sim	27 (90%)
Não	3 (10%)
**Tempo médio da cirurgia até a inclusão no estudo**	8,11 anos
**Tempo Médio de Circulação Extracorpórea**	98,84 min
**Diagnóstico**	
DSA	4 (13,3%)
DSAV	3 (10%)
TGA	3 (10%)
TF	3 (10%)
CA	2 (6,6%)
Insuficiência Aórtica	2 (6,6%)
DSV + DVSVD + EP + Dextrocardia	2 (6,6%)
Outros**	11 (36,6%)

*Gravidade da CC de acordo com o American College of Cardiology.^19^ **Outros: Atresia mitral, EP, VU, DSA+AP, DSV+AP, DSV+PCA, DSV+PCA+EP, DSAV+RVPAT, DSAV+DVSVD, PCA+CA, VU+TGA. DSA: defeito do septo atrial; DSAV: defeito do septo atrioventricular; CA: coarctação da aorta; CC: cardiopatia congênita; DVSVD: dupla via de saída do ventrículo direito; AP: atresia pulmonar; PCA: persistência do canal arterial; EP: estenose pulmonar; VU: ventrículo único; RVPAT: retorno venoso pulmonar anômalo total; TGA: transposição das grandes artérias; TF: Tetralogia de Fallot; DSV: defeito do septo ventricular.

As comparações do grupo CC e controles saudáveis são mostradas na Tabela 3, Tabela 4 e na Figura Central. O grupo controle completou o teste TGlittre-P em um tempo significativamente menor do que o grupo CC. Uma diferença significativa foi encontrada entre as distâncias do TC6M para os dois grupos. No grupo CC, 11 crianças apresentaram dessaturação^29^ nos testes TGlittre-P e TC6M. Dessas crianças, 10 apresentavam CC cianótica e 1 atresia mitral, todas operadas. Observou-se uma diferença significativa entre os grupos nos testes de *sit-ups*, flexões, elevação de tronco e sentar e alcançar. Houve uma diferença significativa entre os grupos em relação às forças de preensão manual da mão dominante e não dominante. Houve uma diferença significativa entre os escores de postura dos dois grupos. Não houve diferença significativa entre os níveis de atividade física dos dois grupos. Os escores de qualidade de vida avaliados pelo PedsQL foram significativamente maiores no grupo controle do que no grupo CC, tanto na forma infantil quanto na forma parental.

**Tabela 3:** – Comparação do grupo CC com controles saudáveis em relação às atividades de vida diária e capacidade funcional

Variável	Grupo CC (n = 30)	Grupo controle (n = 30)	Valor de z/t	Valor de p
**TGlittre-P**				
Tempo de conclusão do teste (min)	3,45 (3,24-4,02)	3,10 (2,57-3,23)	-4,984 ^b^	0,001**
% de Duração	122,67 (113,13-136,04)	104,88 (100,67-113,23)	-4,576 ^b^	0,001**
SpO_2_ pré-teste	98,00 (94,00-99,00)	98,00 (98,00-99,00)	-2,357 ^b^	0,018*
SpO_2_ pós-teste	94,5 (83,25-97,00)	98,00 (97,00-99,00)	-4,700 ^b^	0,001**
∆ SpO_2_	-3,00 ((-9,75) – (-1,00))	0,00 ((-1,25)-0,00)	-4,606 ^b^	0,001**
FC pré-teste	93,86 ± 12,19	93,83 ± 16,69	0,274 ^c^	0,785
FC pós-teste	120,00 (92,75-138,75)	147,00 (136,00-154,25)	4,274 ^b^	0,001**
∆ FC	26,50 (4,50-46,00)	57,50 (36,00-66,25)	-4,237 ^b^	0,001**
% de FC máx.	53,60 ± 15,81	69,14 ± 6,79	-4,947 ^c^	0,001**
**TC6M**				
Distância de Caminhada (m)	514,00 (412,50-566,00)	591,50 (533,00-631,00)	-4,081 ^b^	0,001**
% de Distância	81,63 (62,24-87,23)	90,56 (86,74-100,35)	-4,066 ^b^	0,001**
SpO_2_ pré-teste	98,00 (94,00-98,00)	98,00 (98,00-99,00)	-3,328 ^b^	0,001**
SpO_2_ pós-teste	94,50 (84,75-97,00)	98,00 (98,00-99,00)	-5,264 ^b^	0,001**
∆ SpO_2_	-3,00 ((-10,00) – (-1,00))	0,00 ((-1,00)-1,00)	-4824 ^b^	0,001**
FC pré-teste	92,86 ± 13,53	90,23 ± 14,39	0,730 ^c^	0,468
FC pós-teste	111,43 ± 30,25	139,96 ± 21,18	-4,231 ^c^	0,001**
∆ FC	18,56 ± 29,00	49,73 ± 25,23	-4,440 ^c^	0,001**
% de FC máx.	53,11 ± 14,54	66,73 ± 10,35	-4,176 ^c^	0,001**

b: Teste de Mann Whitney U; c: Teste t de amostra independente; CC: cardiopatia congênita; FC: frequência cardíaca; SpO_2_: Saturação; TC6M: teste de caminhada de seis minutos, p < 0,05*, p < 0,01**

**Tabela 4:** – Comparação do grupo CC com controles saudáveis em relação a aptidão física, postura, força de preensão manual, nível de atividade física e qualidade de vida

Variável		Grupo CC (n = 30)	Grupo controle (n = 30)	Valor de z/t	Valor de p
**Aptidão Física**					
Sit-ups (repetição máxima)		5,00 (0,75-11,25)	15,00 (10,00-21,75)	-4,756 ^b^	0,001**
Flexões de Braço (repetição máxima)		1,00 (0,00-8,25)	5,00 (3,00-7,25)	-2,537 ^b^	0,011*
Elevação do Tronco (distância em cm)		21,00 (18,00-23,25)	25,50 (22,75-27,25)	-2,993 ^b^	0,003**
Sentar e Alcançar (distância em cm)		-10,00 ((-14,25)-1,00)	-1,00 ((-9,00)-1,25)	-2,282 ^b^	0,022*
**Força de preensão manual**					
Dominante (kg)		14,51 (12,13-20,86)	18,82 (15,64-23,58)	-2,029 ^b^	0,042*
Não dominante (kg)		13,60 (11,33-18,48)	18,14 (14,39-21,31)	-2,609 ^b^	0,009**
**Escore de postura**		1,50 (0,00-3,00)	0,00 (0,00-1,00)	-2,862 ^b^	0,004**
**Pedômetro (número médio de passos por dia)**		5.909,71 (3.924,50-9.699,42)	6.566,35 (4.512,28-10.027,60)	-0,857 ^b^	0,391
**PedsQL**					
Escore total	Criança	73,04 ± 14,23	87,24 ± 11,08	-4,313 ^c^	0,001**
	Pais	69,94 ± 14,68	87,17 ± 12,78	-4,847 ^c^	0,001**
Escore de Função Física	Criança	71,04 ± 16,01	91,04 ± 11,54	-5,549 ^c^	0,001**
	Pais	70,77 ± 19,01	91,25 ± 14,50	-4,690 ^c^	0,001**
Escore Total de Saúde Psicossocial	Criança	74,11 ± 14,48	85,22 ± 11,74	-3,264 ^c^	0,002**
	Pais	69,50 ± 14,17	85,00 ± 12,89	-4,430 ^c^	0,001**

b: Teste de Mann Whitney U; c: Teste t de amostra independente; CC: cardiopatia congênita, p < 0,05*, p < 0,01**

## Discussão

Nosso estudo mostrou que as atividades de vida diária, a capacidade funcional, a força de preensão manual, a aptidão física e a qualidade de vida de crianças com CC diminuíram em comparação com o grupo CS. Além disso, elas também apresentaram postura pior do que o CS, mas seus níveis de atividade física eram semelhantes.

Os pesos corporais e os índices de massa corporal do grupo CC foram significativamente menores do que os do CS. As cardiopatias congênitas também são associadas com atraso no crescimento e desenvolvimento devido ao aumento das necessidades de energia e trabalho respiratório, hipóxia, que dificulta a ingestão de alimentos, desnutrição e má absorção.^30^ De forma consistente com nosso estudo, Feldt et al.,^31^ observaram pesos corporais mais baixos, alturas mais baixas e pesos mais afetados pela altura em comparação com crianças saudáveis.

Vinte e sete (27) crianças em nosso grupo CC tiveram cirurgia cardíaca prévia e três não tiveram intervenção cirúrgica. A inclusão de três pacientes que não foram submetidos à cirurgia cardíaca em nosso estudo pode ter melhorado o desempenho do grupo CC, já que as intervenções cardíacas foram associadas a um pior desempenho físico.

Em um estudo que examinou as atividades de vida diária de pacientes com CC complexa, a taxonomia de AVD foi utilizada, e observou-se que as AVDs dessas crianças foram significativamente prejudicadas.^17^ Em nosso estudo, usamos o teste TGlittre-P, um método de avaliação diferente, e obtivemos resultados semelhantes. Usando a taxonomia de AVD, as crianças são avaliadas com base em sua capacidade de realizar 11 atividades, incluindo comer e beber, mobilidade, usar o banheiro, vestir-se, higiene pessoal, cuidados pessoais, comunicação, transporte, compras e limpeza, mas o desempenho da vida diária não pode ser avaliado. Nosso estudo é o primeiro a avaliar as atividades de vida diária em crianças com CC complexa e a usar o teste TGlittre-P, que é um teste de exercício.

O teste Glittre-ADL original é um teste submáximo desenvolvido para avaliar atividades (caminhar, movimentar os membros superiores, sentar-se e subir e descer escadas) que os indivíduos com DPOC frequentemente repetem em suas vidas diárias.^32^ Quanto menor o tempo de conclusão do teste, melhor será o desempenho da vida diária do indivíduo. Martins et al.,^18^ modificaram o teste original de Glittre-ADL para crianças e mostraram que o teste TGlittre-P era válido e confiável para crianças do grupo CS de 6 a 14 anos.^18^ Em estudos realizados em diferentes grupos de doenças, observou-se que o tempo de conclusão do teste dos pacientes é maior em comparação com controles saudáveis.^33,34^ Além disso, Scalo et al.,^35^ relataram que crianças com fibrose cística necessitaram de mais tempo para concluir o teste em comparação com controles saudáveis, mas não houve diferença estatística. Fernandes-Andrade et al.,^36^ avaliaram o teste Glittre-ADL em pacientes com idade entre 18-80 anos com doença cardiovascular e os indivíduos completaram o teste em média em 3,24 min. Em nosso estudo, o grupo controle completou o teste TGlittre-P em 3,10 minutos, e as crianças com CC moderada e grave completaram o teste TGlittre-P em mais tempo (3,45 min). Ao final do teste TGlittre-P, o grupo controle atingiu 69,14% da frequência cardíaca máxima e completou o teste no nível submáximo. Já o grupo CC atingiu 53,60% da frequência cardíaca máxima e completou o teste abaixo do nível da frequência cardíaca submáxima. Além disso, no TC6M, um teste de campo submáximo avaliado em nosso estudo, observou-se que a frequência cardíaca máxima do grupo CC atingiu 53,11%, de forma semelhante ao teste TGlittre-P, e a frequência cardíaca submáxima permaneceu abaixo do nível. Esses dois resultados corroboram um ao outro e podem ser explicados pelas respostas cronotrópicas insuficientes de crianças com CC. O coração menor das crianças resulta em um volume sistólico menor. Por esse motivo, as crianças aumentam principalmente seu débito cardíaco, aumentando a frequência cardíaca para atender à maior demanda por oxigênio durante o exercício. No entanto, a atividade do sistema nervoso parassimpático e simpático, que desempenham um papel importante na regulação da frequência cardíaca em crianças com CC, pode ser afetada por defeitos septais, procedimentos cirúrgicos e condições induzidas por isquemia. Com isso, ocorre a incapacidade de aumentar a frequência cardíaca frente ao aumento da demanda metabólica, ou seja, a insuficiência cronotrópica.^1^ Em nosso estudo, a dessaturação observada em 11 crianças do grupo CC nos testes TGlittre-P e TC6M indica que essas crianças não foram capazes de regular o aumento da demanda metabólica em suas atividades de vida diária. Devido à patologia existente, esta situação é particularmente evidente em crianças com CC cianótica. Além disso, o tempo de conclusão do grupo CC no teste TGlittre-P foi 22,67% maior do que o esperado, e o tempo de conclusão do grupo controle foi 4,88% maior do que o esperado em nosso estudo (tempo médio esperado de conclusão do teste de 2,84 min). O fato de as atividades de vida diária de crianças com CC serem inferiores tanto ao grupo controle quanto ao valor esperado é resultado da incapacidade dessas crianças de aumentar suficientemente sua frequência cardíaca.

De acordo com nosso estudo, a distância do TC6M foi significativamente menor no grupo CC do que no CS. Na literatura, afirma-se que a CC está associada a uma menor capacidade funcional em crianças e adolescentes em comparação com seus pares saudáveis, sendo afetada por uma resposta cronotrópica prejudicada.^1^ Em nosso estudo, de modo semelhante à literatura, observamos que houve diminuição da capacidade funcional, avaliada pela distância do TC6M, em pacientes com CC.

Embora saiba-se que as cardiopatias congênitas afetam as habilidades motoras, poucos estudos avaliam a aptidão física relacionada à saúde dessas crianças. Esses estudos relataram que a aptidão física não foi afetada em crianças com doença de baixa gravidade e diminuiu significativamente à medida que a gravidade da doença aumentava.^5,6^ Em um estudo de Hock et al.,^3^ crianças e adolescentes submetidos à cirurgia de derivação cavopulmonar total foram avaliados por meio da bateria de testes Fitnessgram, e foi relatado que essas crianças apresentaram diminuição da resistência e flexibilidade dos músculos abdominais em comparação com o CS. Também obtivemos resultados semelhantes em nosso estudo. A diminuição da flexibilidade e resistência muscular em crianças com CC pode ser causada por razões multifatoriais, como cirurgias cardíacas prévias, gravidade da doença, comprometimento das atividades físicas e habilidades diárias da criança, superproteção dos pais, recomendações dos médicos para restrição de atividades e o senso de autoinsuficiência da criança em atividades físicas.^2,3,6,37^ Além de todos esses motivos, um estudo mostrou que adolescentes com cardiopatia congênita complexa com IMC baixo apresentaram menor aptidão física.^38^ Em nosso estudo, um dos fatores que causam diminuição da aptidão física no grupo CC pode ser o baixo IMC. A aptidão física é muito importante na avaliação e tratamento desses pacientes; o número limitado de estudos não é suficiente para esclarecer esta questão, sendo necessários mais estudos no futuro.

Foi relatado que a força de preensão manual é um bom indicador da força muscular periférica, bem como da sobrevida na população em geral.^39^ De acordo com dois estudos conduzidos em pacientes com CC leve e moderada, a força de preensão foi semelhante à de controles saudáveis.^5,40^ A força de preensão do nosso grupo composto por casos de CC moderada e grave diminuiu. De forma consistente com nossos resultados, no estudo conduzido por Holm et al.,^2^ verificou-se que a força de preensão estava reduzida em pacientes cardíacos congênitos complexos de 7 a 12 anos. Além disso, resultados semelhantes foram relatados em um estudo abrangente comparando a força de preensão de 385 pacientes cardíacos congênitos com idade média de 27,6 anos com controles saudáveis. Os dados obtidos neste estudo revelaram que a força de preensão foi afetada pelo tipo e gravidade da doença, procedimentos cirúrgicos e intervencionistas anteriores, defeitos residuais e cianose.^41^

Estudos da literatura relatam que escoliose e deformidades cifóticas frequentemente se desenvolvem em crianças com CC submetidas a esternotomia mediana.^12,13^ No entanto, embora intervenções cirúrgicas sejam frequentemente realizadas em crianças com CC moderada e grave, estudos que avaliam a postura são muito limitados.^14^ Nosso estudo é um dos raros exemplos que mostram deterioração da postura na CC. A causa do distúrbio pode ser devido à diminuição da resistência e flexibilidade muscular ou por procedimentos cirúrgicos prévios. Novas pesquisas, incluindo avaliações posturais estáticas e tridimensionais em pacientes com CC, orientarão as práticas de exercícios a serem adicionadas ao conteúdo dos programas de fisioterapia.

Os resultados de estudos relacionados aos níveis de atividade física de crianças com CC em comparação com seus pares saudáveis são contraditórios. O fato de os resultados dos estudos serem diferentes pode ser devido ao resultado da escolha de diferentes métodos para avaliar a atividade física. Estudos usando questionários de atividade física relatados por crianças e seus pais mostram que os níveis de atividade física de crianças com CC são mais baixos do que seus pares saudáveis.^10^ Embora existam diferenças entre os estudos, a maioria deles, especialmente aqueles que usam métodos de medição objetivos, relataram que crianças com CC apresentam níveis de atividade física semelhantes aos de seus pares saudáveis.^5,7-9^ Além disso, foi relatado que indivíduos com CC de gravidade diferente entre as idades de 8 a 19 anos apresentam níveis de atividade física semelhantes aos controles saudáveis, independentemente da gravidade da doença.^7^ Em nosso estudo, enquanto o grupo controle realizou uma média de 7.455 (mediana de 6.566) passos diários, o grupo CC deu 6.825 (mediana 5.909) passos, e os níveis de atividade física dos dois grupos foram semelhantes. Embora a princípio possa parecer promissor que crianças com CC tenham níveis de atividade física semelhantes aos de seus pares saudáveis, é preocupante que crianças na população em geral tenham baixos níveis de atividade física. O vício em jogos de computador, o avanço da tecnologia e a transferência dos ambientes sociais infantis para o mundo virtual estão entre os fatores que levam as crianças a uma vida mais sedentária. Além disso, o período coincidente de nosso estudo com a pandemia da COVID-19 ocasionou a restrição de atividades físicas de todos os indivíduos. No Canadá, observou-se uma diminuição de 21-24% na contagem diária de passos de crianças com CC na fase inicial da pandemia de COVID-19, e a razão para esta diminuição foram as medidas contra a COVID-19.^42^ Considerando que a diminuição da atividade física e o sedentarismo estão associados a todas as causas de mortalidade, medidas globais devem ser tomadas a esse respeito.

Nosso estudo determinou que houve uma diminuição na qualidade de vida do grupo CC de acordo com os escores totais da escala de qualidade de vida relatados por crianças e seus pais. Os resultados do nosso estudo são bastante consistentes com os resultados do estudo em que 1.138 crianças e adolescentes com CC entre 8 e 18 anos de idade, com diferentes gravidades da doença, foram avaliados com o PedsQL.^15^ Os baixos escores de funcionamento físico relatados por crianças e seus pais em pacientes com CC refletem as limitações físicas que enfrentam devido ao comprometimento de seus sistemas cardiovasculares. Além disso, o fato de os escores de qualidade de vida relatados pelos pais em nosso grupo CC terem sido menores do que aqueles relatados pelas crianças provavelmente se deve à atitude superprotetora dos pais em relação à doença de seus filhos. Mais estudos são necessários para explicar essa situação.

A limitação do nosso estudo é que o período em que trabalhamos coincidiu com a pandemia da COVID-19 e medidas que afetam todos os indivíduos foram tomadas. Estudos futuros devem examinar os níveis de atividade física das crianças e de quais parâmetros eles dependem.

## Conclusões

O desempenho das atividades de vida diária, a capacidade funcional, a aptidão física, a força de preensão manual, a postura e a qualidade de vida de crianças com CC moderada e grave diminuíram em comparação com seus pares saudáveis. Crianças com CC devem ser encaminhadas para programas de reabilitação para melhorar seu desempenho físico reduzido. Avaliações de atividades de vida diária, da capacidade funcional, da aptidão física, da força muscular, da postura, da atividade física e da qualidade de vida são fundamentais e orientam a criação de programas de reabilitação para essa população pediátrica.
