# Supplementary material for: Atividades de Vida Diária, Atividade Física, Aptidão Física e Qualidade de Vida em Crianças com Cardiopatia Congênita: Um Estudo de Caso-Controle
Source: Arq Bras Cardiol. 2023 Sep 27;120(9):e20230022. [Article in Portuguese] doi: 10.36660/abc.20230022 (PMC10547433; doi:10.36660/abc.20230022)
Supplement: Translation

# Translation: Activities of Daily Living, Physical Activity, Physical Fitness and Quality of Life in Children with Congenital Heart Disease: A Case-Control Study

**Authors:** Berfin Kişin, Sema Savci, Buse Ozcan Kahraman, Aylin Tanriverdi, Hazer Erçan Bozyer, Halise Zeynep Genç, Mustafa Kir

**Affiliations:** 1 Turkey Dokuz Eylül University – Health Science Institute, Izmir – Turkey; 2 Turkey Acıbadem University - Department of Physiotherapy and Rehabilitation, Istanbul - Turkey; 3 Turkey Dokuz Eylül University – School of Physical Therapy and Rehabilitation, Izmir – Turkey; 4 Turkey Çankırı Karatekin University - Department of Physiotherapy and Rehabilitation, Çankırı – Turkey; 5 Turkey Dokuz Eylül University – Department of Pediatric Cardiology, Izmir – Turkey; 6 Turkey Başakşehir Çam and Sakura City Hospital – Department of Pediatric Cardiology, Istanbul – Turkey

**Keywords:** Heart Defects, Congenital, Physical Fitness, Motor Activity, Activities of Daily Living, Quality of Life, Case-Control Studies

## Abstract

**Background:**

Despite reports of reduced physical fitness in children with congenital heart disease (CHD), no specific performance evaluations for activities of daily living have been conducted.

**Objectives:**

The aim was to compare the activities of daily living, quality of life, posture, physical fitness and physical activity levels of children with CHD with healthy controls (HC).

**Methods:**

The study included 30 children aged 6-14 diagnosed with moderate or severe CHD and 30 age-sex-matched HC. The sociodemographic and clinical data of the participants were recorded. All participants went through several tests, namely the TGlittre-P test for activities of daily living, the 6-minute walk test (6MWT) for functional capacity, the Fitnessgram test battery for physical fitness, the hand dynamometer for measuring grip strength, the pedometer for measuring physical activity, and both the child and parents reported the Pediatric Quality of Life Inventory (PedsQL) for evaluating the quality of life, in addition to posture analyses. Values of p < 0.05 were considered statistically significant.

**Results:**

Individuals with CHD had a longer TGlittre-P test completion time and a shorter 6MWT distance than HC (TGlittre-P: CHD 3.45 [3.24-4.02]min vs. HC 3.10 [2.57-3.23]min, 6MWT: CHD 514.00 [412.50-566.00]m vs. HC 591.50 [533.00-631.00]m). For the CHD group, sit-ups, push-ups, trunk lift, and sit-and-reach test scores within the Fitnessgram battery, grip strength, posture, and quality of life scores were lower than those for the HC group. Physical activity levels were similar in the groups.

**Conclusions:**

The performance of activities of daily living, functional capacity, physical fitness, posture, and quality of life of children with moderate and severe CHD were affected compared to healthy peers.

## Introduction

Congenital heart diseases cover a wide range from minor cardiac defects that are not noticed until adulthood or are detected during vigorous exercise, to serious cardiovascular malformations that can be life-threatening. Studies have documented decreases in functional capacity, motor skills, and peripheral muscle strength in children with complex CHD due to multifactorial causes such as cyanosis, increased pulmonary circulation, cardiac interventions, and multiple diagnoses.^1-3^

Physical fitness and physical activity levels are important predictors of cardiovascular health. Improved physical fitness in the early stages of life is associated with a healthier cardiovascular profile in adulthood.^4^ While very few studies evaluate physical fitness in children with CHD,^3,5,6^ physical activity levels differ between studies.^7-10^

Low levels of physical activity and a sedentary lifestyle have been reported to be associated with postural defects.^11^ Additionally, cardiovascular endurance, muscular endurance, muscle strength, flexibility, and body composition, which are physical fitness parameters, are necessary components to create the ideal posture. Studies in the literature have shown that scoliosis and kyphotic deformities often develop after median sternotomies have been performed on children with CHD.^12,13^ However, although surgical interventions are frequently performed on children with moderate and severe CHD, studies evaluating posture are very limited.^14^

Developments in the healthcare sector have increased the life expectancy of individuals with CHD and, therefore, the importance of quality of life has also increased. Studies evaluating health-related quality of life in patients with CHD emphasize that especially physical health-related quality of life decreases due to disease severity and surgical interventions in these individuals compared to healthy individuals.^3,5,15,16^

Decreased functional capacity and physical fitness can affect activities of daily living. There is a study evaluating activities of daily living (ADL) in children with complex CHD.^17^ However, in this study, only a questionnaire was used instead of a performance test specific to the ADL assessment. For all these reasons, our study aimed at comparing the activities of daily living, quality of life, posture, physical fitness, and physical activity levels among children with CHD and healthy children (HC).

## Methods

We conducted a case-control study on children with CHD. The study protocol was accepted by Dokuz Eylül University Non-Interventional Research Ethics Committee with the approval number 2020/29-58 on 07/12/2020. It was held between November 2020 and May 2022 at Dokuz Eylül University Hospital, Department of Pediatrics, Division of Pediatric Cardiology. Written informed consent was obtained from all participants and their guardians.

The CHD group included 30 children with stable clinical status, aged 6-14,^18^ diagnosed with moderate or severe CHD (Atrial Septal Defect, Ventricular Septal Defect, Atrioventricular Septal Defect, Patent Ductus Arteriosus, Coarctation of Aorta, Aortic Insufficiency, Tetralogy of Fallot, Transposition of the Great Arteries, Double Outlet Right Ventricle, Total Anomalous Pulmonary Venous Return, Single Ventricle, Pulmonary Atresia, Pulmonary Stenosis, Mitral Atresia, Dextrocardia). Using the study of Warnes et al.,^19^ we identified the children with moderate to severe CHD that we included in our study and excluded isolated minor cardiac defects that did not require surgical intervention. Orthopedic or neurological problems affecting the tests, mental or psychological problems, acute infection or general fatigue, cardiac surgery within the last six months, and refusal to participate in the study were determined as exclusion criteria. Healthy controls were selected from healthy individuals who applied to the Department of Pediatrics at Dokuz Eylul University Hospital and had not been diagnosed with any disease. Thirty healthy volunteer children of similar age and sex, non-athletes, were included in the study.

### Activities of Daily Living Assessment

The TGlittre-P test is the pediatric version of the original Glittre-ADL test developed to evaluate individuals’ activities of daily living. It was found valid and reliable in healthy children aged 6-14.^18^

The physiotherapist explained the test to each child, showed it, and asked them to try it. This performance test required the child to complete five laps in the shortest amount of time while carrying a backpack,^18^ weighing between 0.5 kg and 2.5 kg, determined according to age and sex. It was initiated when the child got up from a chair with the soles of their feet touching the ground. Then, they walked for 5 m, went up and down two flights of stairs (17 cm high and 27 cm wide), and walked for another 5 m. Three colored bowling pins that weigh 0.5 kg were removed one by one from the shelf, adjusted at eye level by the child, and were placed back on the shelf while being adjusted according to the umbilicus level, then on the floor, then on the shelf at the level of the umbilicus, and finally back on the shelf at eye level. Returning from the same route and sitting on the chair, the tour ended, and the next tour started immediately. During the five rounds, the children moved the bowling pins with their chosen hand while a pulse oximeter device was attached to the other hand’s index finger. During the test, children were given standard voice instructions such as “sit,” “stand up,” and “keep going.” The expected test time developed by Martins et al.^20^ was calculated and compared with the test performance data of the children.

### Functional Capacity Assessment

The 6-minute walk test was used to evaluate functional exercise capacity. The children were asked to walk as fast as they could down a 30 m-long corridor, using the standard words “you’re doing very well” and “keep going” at the end of each minute. Before and after the test, the heart rate, peripheral oxygen saturation, and distance walked were recorded. In addition, the expected distance walked reported by Geiger et al.^21^ was calculated, and the test performance data of the children were compared and evaluated.

### Physical Fitness Assessment

Sit-ups, push-ups, trunk lift and sit-and-reach tests within the Fitnessgram^22,23^ test battery were used to evaluate children’s physical fitness. The maximum number of repetitions correctly completed was measured for the sit-ups and push-ups tests. The distance in the test position was measured and recorded in centimeters for the trunk lift and sit-and-reach tests.

### Hand Grip Strength Evaluation

Hand grip strength was evaluated using the Jamar hand dynamometer (Model 5030J1, Sammons Preston Rolyan, Bolingbrook, IL, USA).^24^ The children were seated in an upright position. They were asked to squeeze the hand dynamometer with their maximum strength, with the elbow in 90° flexion, the arm close to the body, and the wrist in the neutral position. Measurements were repeated three times for the right and left hands, with an interval of 15 seconds, and the highest values were recorded in kilograms.

### Posture Evaluation

The postural analysis form of Corbin et al.^25^ was used to determine postural disorders. The form is based on identifying postural disorders with posterior and lateral observation and scoring them according to severity (0, none; 1, mild; 2, moderate; 3, severe). It also allows us to classify the postural status according to the total score. Evaluations were made in the standing position without shoes and wearing a thin, comfortable dress suitable for the assessment of posture, and the determined findings were recorded.

### Physical Activity Assessment

Children’s physical activity levels were evaluated using a pedometer (Yamax CW700 Digi-walker Pedometer, Yamax Corp, Tokyo, Japan).^26^ The devices were used to record the children’s normal stride lengths and body weights. Pedometers were attached to their clothes by determining the projection of the kneecap’s midpoint to the pelvis’s anterior surface. The children were asked to use the pedometer continuously for seven days, except for bathing and sleeping hours. After seven days of use, a one-day average step count was calculated.

### Quality of Life Assessment

PedsQL is a 23-item scale including physical, emotional, social and school functionality.^27^ Higher scores indicate a better health-related quality of life for the child. The PedsQL 4.0 Generic Core Scales,^27^ 5-7, 8-12 and 13-18 age child and parent forms were used in our study. The total scale score, physical functionality score, and psychosocial health score consisting of emotional, social, and school functionality scores were calculated.

It was explained to the children that they should say if they felt dizzy, palpitations, chest pain, difficulty breathing or excessive fatigue during all the tests. They could rest and continue or finish the test if necessary.

### Statistical Method

Based on the study comparing the TGlittre-ADL test in chronic obstructive pulmonary patients and healthy controls, the smallest sample size was calculated using the G*Power 3.1 program.^28^ It was calculated that 36 participants, including at least 18 children in each group, should be included in the study, with a calculated effect size of 0.97, an alpha error probability of 0.05, and a power of 80%.

IBM SPSS Statistics (Version 26.0) program was used to analyze the data obtained from the participants. Kurtosis/skewness values, Shapiro-Wilk tests, detrended normal Q-Q plot, and histogram graphs were used to determine the conformity of the variables to the normal distribution. The difference between categorical variables was analyzed by the Chi-square test. The Mann-Whitney *U* test was used to compare the differences between groups for conditions that did not conform to the normal distribution. A t-test for independent groups was used to compare the differences between groups for conditions that conformed to the normal distribution. Categorical variables were expressed as absolute values and percentages. Continuous variables with normal distribution were expressed as mean and standard deviation, and continuous variables with non-normal distribution were expressed as median and interquartile range. Values of p < 0.05 were considered statistically significant.

## Results

The demographic characteristics of the participants are shown in Table 1. There was no statistically significant difference between the CHD group and the control group in terms of age, sex, and height. The body weight and body mass index of the CHD group were found to be lower than the control group. All children were required to attend school remotely due to restrictions imposed by the Covid-19 pandemic during the period when they were included in the study. Consequently, there is no difference between the children’s school attendance status.

**Table 1:** – Demographic characteristics of the participants

Parameter	CHD group (n = 30)	Control group (n = 30)	x^2^ /z-Value	p-Value
Sex Girl/Boy (%)	11/19 (36.7/63.3)	11/19 (36.7/63.3)	0.000 ^a^	1.000
Age (year)	9.50 (8.00-12.25)	9.50 (8.00-13.00)	-0.037 ^b^	0.970
Height (cm)	134.50 (128.50-145.00)	137.00 (130.00-149.25)	-0.710 ^b^	0.477
Weight (kg)	27.50 (25.00-45.00)	36.50 (27.75-52.25)	-2.058 ^b^	0.040*
BMI (kg/m^2^)	16.18 (14.11-20.26)	19.87 (16.71-22.06)	-2.728 ^b^	0.006**

a: Yates’ chi-square test; b: Mann Whitney U test; BMI: body mass index; CHD: congenital heart disease, p < 0.05*, p < 0.01**

The clinical characteristics of the CHD group are shown in Table 2.

**Table 2:** – Clinical characteristics of the CHD group

Type of the CHD	
Acyanotic	20 (66.6%)
Cyanotic	10 (33.3%)
CHD Severity*	
Moderate	16 (53.3%)
Severe	14 (46.6%)
**Surgical Treatment**	
Yes	27 (90%)
No	3 (10%)
**Mean time from surgery to inclusion in the study**	8.11 anos
**Mean Cardiopulmonary Bypass Time**	98.84 min
**Diagnosis**	
ASD	4 (13.3%)
AVSD	3 (10%)
TGA	3 (10%)
ToF	3 (10%)
CoA	2 (6.6%)
Aortic Insufficiency	2 (6.6%)
VSD + DORV + PS + Dextrocardia	2 (6.6%)
Others**	11 (36.6%)

*CHD severity according to the American College of Cardiology.^19^ **Others: Mitral Atresia, PS, SV, ASD+PA, VSD+PA, VSD+PDA, VSD+PDA+PS, AVSD+TAPVR, AVSD+DORV, PDA+CoA, SV+TGA. ASD: atrial septal defect; AVSD: atrioventricular septal defect; CoA: coarctation of aorta; CHD: congenital heart disease; DORV: double outlet right ventricle; PA: pulmonary atresia; PDA: patent ductus arteriosus; PS: pulmonary stenosis; SV: single ventricle; TAPVR: total anomalous pulmonary venous return; TGA: transposition of the great arteries; ToF: Tetralogy of Fallot; VSD: ventricular septal defect.

Comparisons of the CHD group and healthy controls are shown in Table 3, Table 4 and Central Illustration. The control group completed the TGlittre-P test in a significantly shorter time than the CHD group. A significant difference was found between the 6MWT distances of the two groups. In the CHD group, 11 children were desaturated^29^ in the TGlittre-P and 6MWT tests. Of these children, 10 had cyanotic CHD, and 1 had mitral atresia, all of whom had undergone surgery. There was a significant difference between the groups in sit-ups, push-ups, trunk lifts and sit-and-reach tests. There was a significant difference between the groups in dominant and non-dominant hand grip strengths. There was a significant difference between the posture scores of the two groups. There was no significant difference between the physical activity levels of the two groups. Quality of life scores assessed by PedsQL were significantly higher in the control group than in the CHD group in both the child and parent forms.

**Table 3:** – Comparison of the CHD group with healthy controls in terms of activities of daily living and functional capacity

Variable	CHD group (n = 30)	Control group (n = 30)	z/t -Value	p-Value
**TGlittre-P**				
Test Completion Time (min)	3.45 (3.24-4.02)	3.10 (2.57-3.23)	-4.984 ^b^	0.001**
% Duration	122.67 (113.13-136.04)	104.88 (100.67-113.23)	-4.576 ^b^	0.001**
SpO_2_ pre-test	98.00 (94.00-99.00)	98.00 (98.00-99.00)	-2.357 ^b^	0.018*
SpO_2_ post-test	94.5 (83.25-97.00)	98.00 (97.00-99.00)	-4.700 ^b^	0.001**
∆ SpO_2_	-3.00 ((-9.75) – (-1.00))	0.00 ((-1.25)-0.00)	-4.606 ^b^	0.001**
HR pre-test	93.86 ± 12.19	93.83 ± 16.69	0.274 ^c^	0.785
HR post-test	120.00 (92.75-138.75)	147.00 (136.00-154.25)	4.274 ^b^	0.001**
∆ HR	26.50 (4.50-46.00)	57.50 (36.00-66.25)	-4.237 ^b^	0.001**
Max % HR	53.60 ± 15.81	69.14 ± 6.79	-4.947 ^c^	0.001**
**6 MWT**				
Walking Distance (m)	514.00 (412.50-566.00)	591.50 (533.00-631.00)	-4.081 ^b^	0.001**
% Distance	81.63 (62.24-87.23)	90.56 (86.74-100.35)	-4.066 ^b^	0.001**
SpO_2_ pre-test	98.00 (94.00-98.00)	98.00 (98.00-99.00)	-3.328 ^b^	0.001**
SpO_2_ post-test	94.50 (84.75-97.00)	98.00 (98.00-99.00)	-5.264 ^b^	0.001**
∆ SpO_2_	-3.00 ((-10.00) – (-1.00))	0.00 ((-1.00)-1.00)	-4824 ^b^	0.001**
HR pre-test	92.86 ± 13.53	90.23 ± 14.39	0.730 ^c^	0.468
HR post-test	111.43 ± 30.25	139.96 ± 21.18	-4.231 ^c^	0.001**
∆ HR	18.56 ± 29.00	49.73 ± 25.23	-4.440 ^c^	0.001**
Max % HR	53.11 ± 14.54	66.73 ± 10.35	-4.176 ^c^	0.001**

b: Mann Whitney U test; c: Independent sample t-test; CHD: congenital heart disease; HR: heart rate; SpO_2_: saturation; 6MWT: six minute walking test,p < 0.05*, p < 0.01**

**Table 4:** – Comparison of the CHD group with healthy controls in terms of physical fitness, posture, hand grip strength, physical activity level and quality of life

Variable		CHD group (n = 30)	Control group (n = 30)	z/t -Value	p-Value
**Physical Fitness**					
Sit-ups (maximum repetition)		5.00 (0.75-11.25)	15.00 (10.00-21.75)	-4.756 ^b^	0.001**
Push-ups (maximum repetition)		1.00 (0.00-8.25)	5.00 (3.00-7.25)	-2.537 ^b^	0.011*
Trunk Lift (distance in cm)		21.00 (18.00-23.25)	25.50 (22.75-27.25)	-2.993 ^b^	0.003**
Sit-and-Reach (distance in cm)		-10.00 ((-14.25)-1.00)	-1.00 ((-9.00)-1.25)	-2.282 ^b^	0.022*
**Hand grip strength**					
Dominant (kg)		14.51 (12.13-20.86)	18.82 (15.64-23.58)	-2.029 ^b^	0.042*
Non-dominant (kg)		13.60 (11.33-18.48)	18.14 (14.39-21.31)	-2.609 ^b^	0.009**
**Posture score**		1.50 (0.00-3.00)	0.00 (0.00-1.00)	-2.862 ^b^	0.004**
**Pedometer (average number of steps per day)**		5.909.71 (3.924.50-9.699.42)	6.566.35 (4.512.28-10.027.60)	-0.857 ^b^	0.391
**PedsQL**					
Total Score	Child	73.04 ± 14.23	87.24 ± 11.08	-4.313 ^c^	0.001**
	Parent	69.94 ± 14.68	87.17 ± 12.78	-4.847 ^c^	0.001**
Physical Functioning Score	Child	71.04 ± 16.01	91.04 ± 11.54	-5.549 ^c^	0.001**
	Parent	70.77 ± 19.01	91.25 ± 14.50	-4.690 ^c^	0.001**
Psychosocial Health Total Score	Child	74.11 ± 14.48	85.22 ± 11.74	-3.264 ^c^	0.002**
	Parent	69.50 ± 14.17	85.00 ± 12.89	-4.430 ^c^	0.001**

b: Mann Whitney U test; c: Independent sample t-test; CHD: congenital heart disease, p < 0.05*, p < 0.01**

## Discussion

Our study showed that the activities of daily living, functional capacity, hand grip strength, physical fitness, and quality of life of children with CHD decreased compared to HC’s. They also had worse posture than HC, but their physical activity levels were similar.

The CHD group’s body weights and body mass indexes were significantly lower than the HC’s. Congenital heart diseases are associated with delayed growth and development due to increased energy requirements and respiratory workload, hypoxia that makes food intake difficult, malnutrition and malabsorption.^30^ Consistent with our study, in a study by Feldt et al.,^31^ it was shown that children with CHD had lower body weights, shorter heights, and weights affected more by their height than healthy children.

While 27 children in our CHD group had previous cardiac surgery, three had no surgical intervention. Including three patients who had not undergone cardiac surgery in our study may have improved the performance of the CHD group, as cardiac interventions have been reported to be associated with worse physical performance.

In a study examining the activities of daily living of patients with complex CHD, the ADL Taxonomy was used, and it was reported that the ADLs of these children were significantly lower.^17^In our study, we used the TGlittre-P test, a different evaluation method, and obtained similar results. Using the ADL Taxonomy, children are assessed based on their ability to perform 11 activities, including eating and drinking, mobility, going to the toilet, dressing, personal hygiene, personal care, communication, transportation, shopping, and cleaning, but their daily life performance cannot be evaluated. Our study is the first to evaluate activities of daily living in children with complex CHD and to use the TGlittre-P test, which is an exercise test.

The original Glittre-ADL test is a submaximal test developed to evaluate the activities (walking, upper extremity movements, sitting up, and going up and down stairs) that individuals with COPD frequently repeat in their daily living.^32^ The shorter the test completion time, the better the individual’s daily living performance. Martins et al.^18^ modified the original Glittre-ADL test for children and showed that the TGlittre-P test was valid and reliable for HC aged 6-14.^18^ In studies conducted in different disease groups, it has been reported that the test completion time of patients is longer compared to healthy controls.^33,34^ Scalo et al.^35^ reported that children with cystic fibrosis completed the test longer than healthy controls, but there was no statistical difference. Fernandes-Andrade et al.^36^ evaluated the Glittre-ADL test in patients aged between 18-80 years with cardiovascular disease, and individuals completed the test in an average of 3.24 min. In our study, while the control group completed the TGlittre-P test in 3.10 minutes, children with moderate and severe CHD completed the TGlittre-P test in a longer time (3.45 min) than the healthy children. At the end of the TGlittre-P test, the control group reached 69.14% of the maximum heart rate and completed the test at the submaximal level. The CHD group, on the other hand, reached 53.60% of the maximal heart rate and completed the test below the submaximal heart rate level. Additionally, in the 6MWT, a submaximal field test that we evaluated in our study, it was observed that the maximum heart rate of the CHD group reached 53.11%, similar to the TGlittre-P test, and the submaximal heart rate remained below the level. These two results support each other and can be explained by the insufficient chronotropic responses of children with CHD. Small heart size in children results in lower stroke volume. For this reason, children primarily increase their cardiac output by increasing their heart rate to meet the increased oxygen demand during exercise. However, parasympathetic and sympathetic nervous system activity, which plays an important role in heart rate regulation in children with CHD, may be affected by septal defects, surgical procedures and ischemia-induced conditions. As a result, the inability to increase the heart rate against the increased metabolic demand, that is, chronotropic insufficiency occurs.^1^ In our study, the desaturation observed in 11 children in the CHD group in the TGlittre-P and 6MWT tests indicates that these children could not regulate the increase in metabolic demand in their activities of daily living. Due to the existing pathology, this situation is particularly evident in children with cyanotic CHD. In addition, the completion time of the CHD group in the TGlittre-P test was 22.67% longer than expected, and the completion time of the control group was found to be 4.88% longer than expected in our study (expected mean test completion time of 2.84 min). The fact that the activities of daily living of children with CHD are lower than both the expected and control groups is a result of the inability of these children to raise their heart rates sufficiently.

According to our study, the 6MWT distance was significantly shorter in the CHD group than in the HC. In the literature, it has been stated that CHD is associated with lower functional capacity in children and adolescents compared to their healthy peers and is affected by impaired chronotropic response.^1^ In our study, similar to the literature, it was shown that there was a decrease in functional capacity, evaluated by the 6MWT distance, in patients with CHD.

Although it is known that congenital heart diseases affect motor skills, very few studies evaluate these children’s health-related physical fitness. These studies reported that physical fitness was not affected in children with low disease severity, and it decreased significantly as the disease severity increased.^5,6^ In a study by Hock et al.,^3^ children and adolescents who had undergone total cavopulmonary shunt surgery were evaluated using the Fitnessgram test battery, and it was reported that these children had decreased abdominal muscle endurance and flexibility compared to HC. We also obtained similar results in our study. Decreased flexibility and muscle endurance in children with CHD may be caused by multifactorial reasons such as previous cardiac surgeries, the severity of the disease affecting the child’s daily physical activities and skills, parental overprotection, physicians’ recommendations for activity restriction, and the child’s sense of self-insufficiency in physical activity.^2,3,6,37^ In addition to all these reasons, it was reported in a study that adolescents with complex congenital heart disease with low BMI had lower physical fitness.^38^ In our study, one of the factors causing decreased physical fitness in the CHD group may be low BMI. Physical fitness is very important in the evaluation and treatment of these patients; the limited number of studies is not enough to illuminate this issue, and more studies are needed in the future.

It has been reported that hand grip strength is a good indicator of peripheral muscle strength as well as survival in the general population.^39^ According to two studies conducted on patients with mild and moderate CHD, grip strength was similar to that of healthy controls.^5,40^ The grip strength of our group consisting of moderate and severe CHD cases decreased. Consistent with our results, in the study conducted by Holm et al.,^2^ it was found that the grip strength was decreased in complex congenital heart patients aged 7-12. In addition, similar results were reported in a comprehensive study comparing the grip strengths of 385 congenital heart patients with a mean age of 27.6 years with healthy controls. The data obtained from this study revealed that grip strength was affected by the type and severity of the disease, previous surgical and interventional procedures, residual defects and cyanosis.^41^

Studies in the literature report that scoliosis and kyphotic deformities often develop in children with CHD who underwent median sternotomy.^12,13^ However, although surgical interventions are frequently performed on children with moderate and severe CHD, studies evaluating posture are very limited.^14^ Our study is one of the rare studies showing deterioration of posture in CHD. The cause of the disorder may be due to decreased muscle endurance and flexibility or previous surgical procedures. Further research, including static and three-dimensional posture assessments in patients with CHD, will guide the exercise practices to be added to the content of physiotherapy programs.

Results of studies related to physical activity levels of children with CHD compared to their healthy peers are contradictory. The fact that the results of the studies are different may be due to the result of choosing different methods to evaluate physical activity. Studies using physical activity questionnaires reported by children and their parents show that the physical activity levels of children with CHD are lower than their healthy peers.^10^ Although there are differences between studies, most of them, especially those using objective measurement methods, have reported that children with CHD have similar physical activity levels to their healthy peers.^5,7-9^ In addition, it has been reported that individuals with CHD of different severity between ages 8-19 have similar physical activity levels to healthy controls, regardless of disease severity.^7^ In our study, while the control group took an average of 7455 (median 6566) daily steps, the CHD group took 6825 (median 5909) steps, and the physical activity levels of the two groups were found to be similar. Although it may seem promising at first that children with CHD have similar levels of physical activity as their healthy peers, it is worrisome that children in the general population have low levels of physical activity. Computer game addictions, the development of technology, and the transfer of children’s social environments to the virtual world are among the factors that lead children to a sedentary life. In addition, the coinciding period of our study with the Covid-19 pandemic caused the restriction of physical activities of all individuals. In Canada, it was stated that there was a 21-24% decrease in the daily step count of children with CHD in the early phase of the Covid-19 pandemic, and the reason for this decrease was due to Covid-19 measures.^42^ Considering that decreased physical activity and sedentary life are associated with all causes of mortality, global measures should be taken in this regard.

Our study determined that there was a decrease in the quality of life of the CHD group according to the total quality of life scale scores reported by both children and their parents. The results of our study are quite consistent with the results of the study in which 1138 children and adolescents with CHD between ages 8-18 with different disease severity were evaluated with PedsQL.^15^ The low physical functioning scores reported by both children and their parents in patients with CHD reflect the physical limitations they face due to their impaired cardiovascular systems. In addition, the fact that the quality of life scores reported by the parents in our CHD group was lower than that reported by the children is likely due to the parents’ overprotective attitude against their child’s disease. Further studies are needed to explain this situation.

The limitation of our study is that the period we are working on overlaps with the Covid-19 pandemic, and measures that affect all individuals have been taken. Future studies should examine children’s physical activity levels and what parameters it depends on.

## Conclusions

The performance of activities of daily living, functional capacity, physical fitness, hand grip strength, posture, and quality of life of children with moderate and severe CHD decreased compared to their healthy peers. Children with CHD should be referred to rehabilitation programs to improve their reduced physical performance. Assessments for activities of daily living, functional capacity, physical fitness, muscle strength, posture, physical activity, and quality of life are very important and guide the creation of rehabilitation programs for these children.
